# The transformative role of interaction rituals within therapeutic communities

**DOI:** 10.1111/1467-9566.12773

**Published:** 2018-06-29

**Authors:** Jenelle M. Clarke, Justin Waring

**Affiliations:** ^1^ Business School North University of Nottingham Nottingham UK

**Keywords:** Emotions, mental health and illness, mental health services, rituals, social support

## Abstract

Mental health settings are fraught with emotion as clients address difficult life experiences and relational patterns. Clients spend a substantial amount of time together outside of structured therapy, but little is known about how these moments are potentially therapeutic, especially as sites of emotional change. We draw on interaction ritual chain theory to explore how negative emotions in situations outside of formal therapy can be transformed into positive emotions and facilitate personal change. The research is based upon a narrative ethnography of two therapeutic communities for individuals with a diagnosis of personality disorder. Despite the presence of negative transient emotions in these rituals, clients experienced positive feelings of solidarity and belonging, and the majority of clients reported increased feelings of confidence and positive change. Conversely, dynamics between clients showed clients were not always supportive of one another and at times, could exclude others, resulting in isolation and alienation. We argue interactions that generate feelings of inclusion or exclusion over time are a key component in whether clients gain positive or negative emotional feeling and experience personal change.

## Introduction

Individuals attending inpatient and outpatient mental health services spend much of their time interacting outside of structured of therapy, such as smoking breaks and mealtimes. Research has identified these interactive situations as sites of intense emotion and conflict, especially between clients and staff (c.f. Bloor *et al*. 1988, Goffman 1961, McKeganey and Bloor 1987, Quirk *et al*. 2006, Wood *et al*. 2013). Despite being integral to the social organisation of mental health services, little research has considered whether, and how, social encounters outside of therapy can be therapeutic.

In this paper, we draw on Collins's (2004) interaction ritual chain (IRC) theory to understand how everyday social interactions outside of formal therapy can facilitate transformative personal change. Instead of focusing on clients’ psychiatric condition, we place the interactive situation, or ritual, at the centre of analysis. Following Collins, we see an interaction ritual as a ‘mechanism of change’ (Collins 2004: 43), with the potential to influence interpersonal relationships and patterns of group interaction. We are particularly interested in IRCs occurring outside of formal therapy and how these enable the expression of shared emotional experiences, feelings of group belonging, and therapeutic change. To date, IRC theory has not been widely applied to mental health services and, we suggest, the theory does not fully explain how interactions with intense negative emotions generate positive feelings of solidarity and confidence.

The research was undertaken in two therapeutic communities (TCs) for individuals with a diagnosis of personality disorder (PD). Unlike acute ‘hospital’ wards, TCs consider all aspects of community life potentially therapeutic, including encounters outside of structured therapy (Spandler 2009). Whilst Castillo (2003) argues a sense of belonging within TCs is crucial for promoting positive change, it is unclear how communities foster and sustain this sense of belonging, especially during informal times. This paper draws on IRC theory to explain how seemingly mundane interactions outside of structured therapy can facilitate a sense of belonging and therapeutic change. It is unknown, for example, how interactions in such mental health settings transform negative emotions into positive change. We expand IRC theory to consider the role of negative emotions within mental health settings and highlight the importance of belonging in transforming negative feeling. Our study indicates that inclusion or exclusion of members determines whether individuals gain positive or negative emotional feeling from an interaction.

## Interaction ritual chain theory

Interaction ritual chain (IRC) is a microsociological theory that places everyday social interactions at the centre of analysis. The conceptualisation and analysis of a ritual can be traced to Durkheim (1912/2001) on the macro level, Goffman (1967) on the micro level, and is more recently developed by Collins (2004). Broadly, rituals are a form of social interaction with relatively stable elements through which symbolic meanings and norms are transmitted and reinforced, shared identities are fostered, and a sense of belonging promoted. Rituals are sociologically significant because they represent an interactive medium that link individual agents to wider social and cultural structures.

For Collins (2004), rituals consist of four main ingredients: co‐presence, barrier to outsiders, mutual focus, and shared mood; so, rituals involve social actors interacting together within a shared spatial and temporal situation, where there is a boundary to legitimate participation, and where participants have a shared sense of purpose and emotional experience. This emotional state is central to members’ conscious awareness of themselves and their sense of belonging (Summers‐Effler 2004).

Successful rituals generate four main outcomes: ‘solidarity’ or the feeling of belonging to the group; ‘emotional energy’ or long‐lasting and rewarding feelings; ‘symbols’ including the objects, ideas, gestures, words or images representative of the group; and the ‘sense of morality’ members experience from belonging to the group (Collins 2004: 49). Of particular importance is emotions. How emotions are defined, and how they relate to health and illness, have a long and complex history (c.f. Freund 1990, Henckes and Nurok 2015, Turner and Stets 2005). In IRC theory, emotions are not rigidly defined, or indeed the primary focus of analysis; instead it places emphasis on ‘emotional energy’ (Collins 2004). Whilst it recognises that transient emotions are dynamic and changeable, IRC is primarily concerned with how such emotions impact subsequent interaction as a form of emotional energy (EE) (Collins 2014). Collins (2004: 108) distinguishes between ‘high’ and ‘low’ EE. High EE includes feelings such as ‘confidence’ and ‘enthusiasm’ that fosters further interaction; whereas low EE is related to feelings of despair and isolation, and can lead to social alienation. ‘High’ and ‘low’ EE have similarities with Freund's (1990) analysis of Buytendijk's (1950) modes of feeling. Freund (1990: 461) notes that ‘unpleasant’ feelings are represented by ‘resistance’ in interactions with others, injury, ‘being subdued’ or a loss of self. Conversely, ‘pleasant’ feelings are ‘a felt sense of unity between self and other’ experienced as a ‘surging outward or movement outward of one's existence’, akin to the ‘energy’ that Collins describes.

Collins extends the work of Durkheim and Goffman to explain how rituals have a past, present and future, linked together through ‘chains’. As Collins (2004: 44) contends, ‘situations generate and regenerate the emotions and the symbolism that charge up individuals and send them from one situation to another’. However, rituals that fail to charge up individuals can result in a lack of commitment to the ritual and community. Individuals are more likely, therefore, to repeat those rituals that provide the highest emotional reward of EE and an increased sense of social connection (Collins 2004). As noted above, the focus of this paper is the potential role for negative transient emotions to also create and sustain feelings of group belonging.

The strength of Collins’ contribution to IR theory is the recognition that interactions are temporally linked and that emotions play an explicit role in shaping everyday social life. Through IRC theory, he implicitly links high EE with hope and low EE with despair, arguing high EE provides individuals with an interactional trajectory or motivation for action. As individuals accumulate an interactional history, their expectations of social interactions form, setting in play a pattern of interaction. If individuals’ interactional histories are marked by abuse or neglect, their expectations of future encounters may be negative, potentially leading to despair or isolation (Summers‐Effler 2004). In contrast, a ‘successful buildup of emotional coordination’ can lead to a shared feeling of ‘solidarity’ (Collins 2004: 108). Both the history of, and motivation for, interaction have links with Freund (1990: 461) who theorises that emotions can be empowering or disempowering, and that these feelings are directly related to ‘conditions of existence encountered throughout one's biography’. Collins thus appears to view EE in relation to absence and presence of long‐term emotion, rather than the affective character of the transient emotion (positive and negative). Some ambivalence remains as to exactly how transient negative emotions can generate high emotional energy.

Researchers have applied Collins's framework in diverse settings, elaborating the relationships between EE and belonging (c.f. Goss *et al*. 2011, Heider and Warner 2010, Henckes and Nurok 2015, King 2006, Olitsky 2007, Rossner 2013, Summers‐Effler 2010, Vertesi 2012). For example, Szymczak (2015) and Hallett (2003) show how individual actions are partly shaped by previous IRCs, strengthening Collins's assertion that interactional histories influence expectations for future interactions. Summers‐Effler (2004) further argues individuals’ negative expectations of interactions can positively change if new positive interactions challenge their expectations. However, Turner and Stets (2005) and Boyns and Luery (2015) have criticised Collins for an underdevelopment of emotional energy (EE). The application of IRC theory within the sociology of health and illness has tended to focus on healthcare workers (Henckes and Nurok 2015, Szymczak 2015). For instance, Henckes and Nurok (2015) highlight how emergency professionals feel a sense of belonging and solidarity through sharing intense emotions. There remains, nonetheless, little research applying IRC theory to consider the interactions between service users. By applying IRC theory to mental health environments, we attempt to further develop EE by examining how feelings of belonging and confidence can occur through negative transient emotions in everyday encounters and highlight some of the complexities and limitations of rituals to promote positive change.

## Therapeutic communities

Mental health services have long been a focus for microsociological analysis, as exemplified by Goffman's (1961) *Asylums*. Clinical and non‐clinical situations are rich in social interactions that contribute to the transformation of social roles, the sense of self and psychological wellbeing. Our study focuses on therapeutic communities (TCs), as a mental health service that emphasises a ‘social’ approach to mental health, over a ‘bio‐medical’ model. Adult democratic TCs in the UK have come to specialise in the treatment of personality disorder (PD), which is often characterised by symptoms including emotional deregulation, self‐harm, and disruptive relational patterns (Spandler 2006, Stalker *et al*. 2005). Categorisation, diagnosis, treatment and even the notion of ‘personality disorder’ have been contested, including within the sociology of health and illness (c.f. Manning 2000, Jutel 2009, Pickersgill 2011, Pilgrim 2001, Stalker *et al*. 2005). Through their focus on the social, TCs seek to challenge the medicalisation of PD.

TCs advocate a focus on exploring difficult relational patterns, recognising that ‘unhelpful’ social relationships can contribute to mental distress, and they value positive relational networks to restore individual mental health (Boyling 2011). Of relevance for this paper, those with a diagnosis of PD have often experienced negative interactions including trauma, neglect and abuse. Solidarity amongst TC members is therefore especially important for promoting belonging and attachment as a basis for positive psychological change (Castillo *et al*. 2013, Haigh 2013).

Alongside structured group and individual psychotherapy, TCs explicitly view times outside of formal therapy as contributing to personal change through ‘living learning’ (Haigh 2013). Boundaries between ‘formal’ and ‘informal’ therapy can be difficult to distinguish, yet the literature acknowledges the distinction and importance of these times. For example, Whiteley (2004: 243) notes that whilst therapeutic insights and transformations could happen in ‘any’ therapeutic setting ‘slightly more of these important and beneficial incidents took place within the community boundaries but outside the formal therapy groups’. These changes can be manifest through feelings of hope, increased self‐understanding, enhanced interpersonal relationships, feelings of mutuality, and improved behavioural patterns (Haigh 2013, Mahony 1979).

Despite the acknowledged importance of encounters outside of structured therapy, few studies explore what occurs during these times. Ethnographic accounts are useful for understanding the principles of TC work (Bloor *et al*. 1988), staff and client teamwork (McKeganey and Bloor 1987), the tensions between TC principles and prison environments (Rawlings 1998), and issues of power (Bloor 1986, Freestone 2005). With the exception of Freestone (2005), however, non‐therapy times are not the explicit focus of such ethnographic research. Knowledge about the importance of these times therefore remains underdeveloped. Although previous research shows everyday interactions in TCs as highly emotive, it is not explained how these times can be therapeutic and how negative emotions can be transformed into positive feelings. Given that TCs explicitly value interactions outside of structured therapy, they are an ideal context to apply IRC theory. We therefore explore how IRCs outside of therapy can produce feelings of belonging and solidarity, even with strong negative feeling, and question the limitations of interactions to promote therapeutic change.

## Study design

The paper is based upon narrative ethnographic research within two TCs. Narrative ethnography focuses on treating the written text as a story (Davies 2008), where the researcher steps into pre‐existing institutional and individual narratives (Bruner 1997), and where the interplay between institutions and episodic interactions determines what narratives are valued and recounted (Baldwin 2005, Gubrium and Holstein 2008).

The study was undertaken with two purposefully selected TCs, each accredited by the Community of Communities (the quality assurance project of the Royal College of Psychiatrists 2018), and addressing the needs of adults with a diagnosis of personality disorder. Their names are anonymised as ‘Powell’, an independent, charitable residential community for women, and ‘Hawthorne’, an NHS day community for men and women; and both offer length of stay between 8–12 months. With the exception of Bloor *et al*. (1988), most ethnographic research within TCs has been single site. Replicating the breadth and depth of Bloor *et al*.'s (1988) ten‐year study is extremely difficult. Nonetheless, this research draws on their approach to explore the common instances of ‘interconnectedness’, ‘relationships’ and ‘interaction rituals’ within and between research sites (Hannerz 2003: 207). The study received favourable ethical approval through standard NHS Research Ethics.

Through written letter, Clarke invited Powell and Hawthorne to take part in the study, and conducted preliminary visits to outline the study purpose to all members. Community members were supportive of the study and agreed to take part. Data collection began in November 2012 and concluded in October 2013. Clarke conducted participant observation for four months at each community, totalling 746 hours; carried out 21 narrative interviews with clients and former clients exploring their experiences of life in the communities; and conducted seven semi‐structured interviews with staff members focusing on contextual information such as the history, values and organisation of the community. Observations did not look at clinical interactions or therapeutic sessions, but the times outside of structured therapy such as community meetings, meal times, smoking breaks, trips to town and shops, and, other informal times in the evenings, nights and weekends. From the outset staff and clients were welcoming to Clarke, inviting her to take part in activities, such as smoking or coffee breaks, and over time, high degrees of rapport and openness was built with members of both communities, supporting closer participation and understanding of group interactions in non‐therapeutic settings. Fieldnotes were written in the field and away from the communities, providing an opportunity for further reflection. With the exception of one client, all interviews were audio recorded and transcribed verbatim.

### Analysing everyday inter‐’action’ rituals

Interpretative data analysis developed a detailed narrative ethnography of the IRCs of both TCs. Data analysis followed in the abductive tradition of relating emergent empirical data back to sensitising concepts and assumptions with the intention of extending, clarifying or challenging pre‐existing theories, while concurrently relating theories back to the empirical findings to inform subsequent data analysis and theming (Tavory and Timmermans 2014).

Clarke first carried out open coding of fieldnotes during data collection to identify emergent themes and inform on‐going enquiries. As data collection progressed these initial codes were refined through constant comparison (Corbin and Strauss 1991) and applied to all data using nVivo (QSR International, Brisbane). Throughout coding consideration was given the sensitising concepts related to rituals (Goffman 1967) and, specifically IRCs (Collins 2004), as described above. As empirical (first order) codes were further analysed, categorised and themed, more explicit consideration was given to Collins's (2004) analytical criteria with the intention of identifying and categorising the different IRCs found within each TC. Waring was involved in these later stages of analysis to offer interpretative clarifications, analytical comparison between TCs, and to explore where emergent findings related to, and also extended, existing theories. Data analysis led to the identification and characterisation of many distinct rituals: 57 (Powell) and 45 (Hawthorne), 22 of which were common to both communities. These rituals were further analysed and categorised in the light of work by Chapple (1970), Collins (2004), Helman (2007), Mandelbaum (1959), Scheff (1977) and Summers‐Effler (2006), including: Inclusion, Exclusion, Reinforcement, Anti‐Group and Transitional (Table 1) (Clarke 2015).

**Table 1:** Summary of common interaction rituals within Therapeutic Communities

Inclusion	Exclusion	Reinforcement	Anti Group	Transitional
meal times	meal times	meal times	smoking breaks	informal social time
smoking breaks	smoking breaks	emergency meetings	arriving late	smoking breaks
informal social time	informal social time	reviews	leaving early	community meetings
emergency meetings	crisis texts			crisis texts
community meetings	reviews			

Inclusion and Exclusion referred to interactions that were inclusive of all or exclusive of some; Reinforcement rituals strengthened group expectations; Anti‐Group were interactions working against TC values; and Transitional were interactions linking one ritual to another, for instance a smoking break linking lunch and an afternoon meeting. Analysis finally centred on emotional energy, especially those rituals that contained intense, negative emotions, yet still appeared to generate solidarity. Three themes became clear, including how negative transient emotions work within rituals, how negative transient emotions are transformed in to EE and the limitations of IRCs to facilitate personal change. The paper focuses on those rituals that exemplify the role of EE and solidarity, including meal times, smoking breaks, informal times, emergency meetings, reviews and crisis texts.

## Findings

### Inclusivity within rituals: solidarity through negative transient emotions

Everyday life within the TCs could be emotionally demanding, what some clients described as a ‘battle’. A shared sense of solidarity and trust was crucial to fostering an inclusive environment for clients to disclose and address distress. For example, solidarity was often nurtured and reinforced through mutual experiences of mental distress, and observed through relatively mundane rituals, such as break times:

Anna, Alison, Erica, Andrea and I chat together in the lounge. Conversation turns to being ‘normal’ as Erica brings it up. Anna says, ‘You know what a former client member says?’

Erica nods and replies, ‘Normal is just a cycle on a washing machine’.

‘What? I don't get it’, states Andrea. […]

‘No one is normal’, explains Anna.

Erica then talks about other people's expectations of her and wanting to ‘just feel normal’.

Alison glances up from her phone and asks, ‘And where has trying to live up to other people's expectations got you?’

Erica immediately responds loudly with a grin and her arm raised in the air, ‘In a mental institution!!’ Everybody laughs. (Powell, Day 39, 12‐13/02/13, waking night shift)

Establishing solidarity could be challenging, especially with so many negative emotions present in the TCs. During his interview, Brian from Hawthorne reflected:

I've seen a lot of arguments (slight laugh) in my time here. A *lot* of arguments. People walking out, people almost like come to fist fights, throwing stuff, yeah, I've seen that a lot.

Many clients reported feelings of isolation and loneliness before coming to the communities, often due to difficulties in personal relationships. Reprising these negative feelings had an explicit therapeutic role for exploring how unhelpful experiences in the past shape unhelpful patterns of relating in the present (Spandler, 2009). As Matthew, a staff member at Powell explained, ‘When you start working on the past, it starts uncannily to play out in the present in the community’. Part of this process involved encouraging negative emotions to surface during interactions so they could be explored as an inclusive community, founded on trust and shared understanding.

At times, the negative emotion itself, such as a mutual dislike for certain groups, could establish solidarity. For example, several clients at Powell did not like art therapy, which occurred once a week, and would frequently complain about it during break time rituals. The opportunity to openly complain was itself premised on a shared (negative) emotion, and further reinforced a sense of solidarity:

Between groups, I go in to the lounge. Erica is working on her puzzle and Julie is on the sofa with Jacob (staff). Anna comes in and sits on another sofa […]. They talk about art therapy. No one wants to go to art. I ask some questions about it and they explain that it's like free association, they walk in and can create anything they want using anything that is available. Julie says the problem is that ‘my head is always blank’. She says, ‘I'll just do another tree’. Erica picks her up on her ‘negative tone’ […]. She says trees are absolutely fine and says that she could also draw a Christmas tree, a flower, etc. (Powell, Day 16, 13/12/12)

Meal time rituals could be particularly difficult, as some clients struggled with disordered eating; nonetheless these rituals provided a foundation for solidarity and inclusivity despite their challenges. Clients in both communities spoke openly with each other about their struggles with food. Though physically present at the table, some clients would withdraw from the conversation, watch others eat, or pick at their food. In response, other clients found ways to offer support, often based upon shared experience:

Conversation around the table feels up beat. There is talk about Margaret's (staff member) dog, cooking, food, etc. Andrea eats very, very slowly whilst staring intently at those around the table. She has a napkin in front of her with writing on it – ‘I DO deserve to eat’. I later learn that Tessa, who is also very open with clients about her struggles with eating, wrote it for her. Andrea has voices telling her that the food is poison and that she doesn't deserve to eat. Yesterday Tessa had suggested cheerleading statements at dinner reminding her that she deserves to eat. (Day 35, 06/02/2012)

Importantly, because rituals are linked together through chains, what happens in one interaction can affect what happens in subsequent interactions. Here, Tessa's encouragement of Andrea the day before was continued in the next meal time. The support was unbroken.

To further explore the role of negative transient emotions in TCs, we draw on the ritual of ‘reviews’ at Hawthorne. Reviews were a reflexive group exercise initiated whenever clients broke a community boundary, which included refraining from self‐harm, abstaining from drugs, phoning in absences, and treating members with respect. They varied in format and duration according to the severity and frequency of the boundaries broken, and whether the individual was already ‘on review’. Clients would then meet with another client mentor to reflect upon what led to the boundary break. Missing a meeting with a mentor meant the review would be extended. ‘Going on review’, and how reviews were managed, would often provoke strong emotional responses.

The observation extract below highlights the response of two clients during a community meeting, and immediately after the meeting, over the issue of missed mentor meetings after two clients, Christopher and Jessie, were required to make‐up their missed meetings:

[During the community meeting] Christopher is angry, shaking his head and speaking loudly about not being supported by the community. Jessie nods and does not complain.

The [community] meeting finishes and I wait to go outside with the smokers. Christopher immediately starts to complain about the process of reviews and Abby snaps back at him, saying, ‘It's your responsibility to make sure you get it done!’

‘What, so I miss a few days, the community thinks I need extra support but *I* have to seek out the support?!’ he replies in a raised voice. ‘Fuck it, I ain't doing it’, he adds. […] When we return inside from the smoking break, Christopher is still unhappy about it but is chatting with the others. (Hawthorne, Day 17, 11 July 2013)

The exchange between Abby and Christopher was energetic, not flat, as they disagreed with each other. Rather than withdrawing or acting excluded, Christopher continued to let others know he was unhappy with the process. Despite the disagreement, the ritual, and the ritual chain, did not fracture but resulted in subsequent interaction. Through expressing negative emotions, including challenging each other, clients found an opportunity to share their experiences with one another.

### Transforming negative transient emotions in to high EE

Whilst interactions in the TCs could generate intense negative emotions, these feelings appeared at times to be transformed in to feelings of belonging and confidence, or high EE. We observed this transformation during some emergency meeting (EM) rituals at Powell. These *ad hoc* meetings were convened for clients who self‐harmed, were feeling distressed, or struggling to commit to their safety. The meetings provided an opportunity to share feelings and seek support. Members would describe their struggles and offer suggestions for how to help others manage their feelings of despair, anxiety, and shame and ultimately to feel safe. Whilst staff offered their perspective, clients chaired the meeting and kept records of conversations.

Although clients frequently described EMs as emotionally draining, they also spoke positively about their role within the TC. In particular, some saw these interactions as particularly inclusive for newer members:

Tessa states EMs are a really good way to feel included in the community. They explain when you are new, being part of an EM and helping someone through a crisis can really make you feel part of the TC. (Powell, Day 31, 29 January 2013)

Summers‐Effler (2010: 118–120) writes that group values and the ‘emotional ideology’ of a community cannot be taught; rather they have to be lived as they are ‘embodied’, not ‘discursive’. For the TCs, such highly emotional meetings were a way for new members to become part of the emotional history of the community. Importantly, they generated feelings of belonging that superseded the negative emotions that triggered the initiation of these rituals.

Looking closer at the experiences of newcomers, negative feeling could often help the lay foundations for shared experience and a sense of belonging. During one interview, Anna explained her reaction to her first involvement in an EM:

And then we sit in a meeting and we're asking her all these questions and I kind of wanted to say just leave her the fuck alone, she can't think straight. […] You're asking people these really important questions when they're clearly not in a place where they can answer it. […] But within 10 minutes, she was sat in the bay of the window with her headphones on, still upset but contained and able to be safe. And I just thought, God, this is really weird. Because all through the meeting I was just thinking, what the hell? And then you're like oh, I don't really get it but it does seem to work somehow. […] but I think it was watching somebody struggle […] I really connected with [her] right from the off. And I think it was that sense of nobody can help me. I think that's what I got from her, the fact that she couldn't imagine there would be anyway that anybody could ever help her. And it was her job to do it alone, which is kind of how I feel.

The twice daily community meeting rituals represented another, less stark, interaction ritual where clients could share their feelings though reciprocal self‐disclosure. Despite negative emotions, these interactions could facilitate opportunities for support and feelings of belonging. Below, Abby recounts a telephone conversation with her mother where she disclosed she had been sexually abused by a family member:

Abby explains that eventually she wants to have the face‐to‐face conversations with her mum about it, that yesterday for her was just the start. Abby says that she could have only done that yesterday, disclose so much and talk it through in group, due to the ‘community holding my hand over the past year’.

Stephen (staff member) says he feels pleased for Abby and for the community for helping her […]. ‘I feel like applauding you, Abby, and the community for the work you all did in helping you work through this’. […]

Kevin then says that ‘everybody should do what Abby did […] We've all got stuff we need to talk about’. He states that while he could not relate to the sexual abuse, a lot of the other stuff she shared did touch on things for him that he knows he needs to explore. (Hawthorne, Day 26, 30/07/13)

As this extract suggests, it was the act of disclosing, rather than the specific character of the experience being disclosed that seemed to resonate with others and encourage further sharing.

Through participating in everyday rituals, the transformation of negative transient emotions into feelings of inclusion and solidarity provided the foundations for more positive emotional and personal change. One of the key theorised outcomes of IRCs is the feeling of confidence. Clients were not asked about ‘confidence’ during the interviews, yet 16 of 21 clients referred directly or indirectly to feeling more confident as a result of their time in the TCs:

I have become more confident in myself than I was when I came in. (Alison, Powell)

It [the TC] gives me hope that I can change the problems that I brought. (Amy, Powell)

I'm starting to like myself bit more than I did. […] Even if it's the smallest bit, I think liking myself is a big achievement. (Jessie, Hawthorne)

The majority of clients interviewed reported positive feelings about themselves and their capacity to manage unpleasant feelings as a result of being in the community. This suggests clients were redefining their definitions of social expectations and their sense of identity based upon new (positive) emotional meanings. That is, individuals used their previous feelings about past encounters to find a sense of belonging and solidarity with others who had similar experiences, or wished to similarly express their own histories.

### Exclusivity within rituals: negative transient emotions reinforcing low EE

However, not all clients reported feelings of increased confidence, and some discussed feelings of marginalisation from the group. At times rituals appeared to fail, sometimes abruptly. For instance, one community meeting ritual at Powell ended when a client began screaming and then refused to speak. After clients’ repeated attempts to assist her, staff members ended the meeting to attend to the client. Other times, rituals could become more exclusive, leaving some clients feeling awkward, hurt and frustrated. These types of issues were sometimes observed within the smoking breaks. Such breaks provided an opportunity for clients to articulate supplementary views about their experiences and feelings outside of formal therapy sessions. They were also used to gossip about other clients or staff. The following extract is of a smoking break ritual where clients reflected on, and joked about, a heated discussion between two clients during a community meeting related to the use of crisis texts, where one client (Robert) shared his views of another (Jessie) in a message to all community members, but excluded Jessie from the text.

I head outside with the smokers and we stand around in a circle, chatting. There is much joking around between Daniel and Abby. She sends him a text, just him, but she shows us what she sent. She calls him ‘cheeky’ in the text. He jokes that she hasn't included everybody. Evan says they need a crisis meeting because she didn't include everyone. […] Jessie shifts from foot to foot, but doesn't say or do anything. They (Daniel, Evan, and Abby) continue to joke about sending texts, excluding each other, and feeling left out. Lauren says nothing. Neither does Jessie.

We start to walk back in and Lauren and Jessie start walking fast, ahead of the rest of us, their heads together, talking. ‘Shit!’’ says Daniel. ‘I hope Jessie doesn't think we were making fun of her’. Evan says, ‘Well hopefully she knows we were just blowing off steam, our way of dealing with a tense situation’ (but how would she know this unless they told her?). (Hawthorne, Day 17, 11 July 2013)

Here, the residue from the previous interactions, namely Jessie being initially left off the crisis text and the heated emotions discussing the text, spilled over into the smoking break. As interactions like crisis texts and community meetings were often emotive, smoking breaks were important for ‘blowing off steam’, as described by Evan, but in this case the joke was interpreted by Evan and Daniel as exclusionary and at the expense of other clients.

Feelings of exclusion could also occur from leaving left out from social activities. At Powell, client member Tessa self‐harmed with a razor and during the subsequent Emergency Meeting ritual, explained that it stemmed from feeling excluded:

Tessa comes in, sitting next me on one of the sofas. She is trembling […], sitting with her legs pulled up to her chest. Her sleeves are long, covering her freshly bandaged wrists. […] Anna questions what triggered it and Tessa explains she felt unwanted and unloved by people here and by her family. She says that after the community meeting she went into her room and did some writing. She could hear people laughing outside her door and she felt excluded. She also felt excluded by not going into town with the others (it is unclear whether she was invited or not). Anna asks her about feeling unwanted by people here and several clients say how much they care about her and love her. Martha says the place could not run without her. Ericka adds she felt sad Tessa was feeling like this and was worried she did not say anything earlier. (Powell, Day 2, 17/11/2012)

Through the lens of IRC theory, Tessa's actions could be interpreted as a form of isolation and despair as she sat in her room on her own, taking out her negative feelings against herself (Summers‐Effler 2002). The chain of events began with her feeling excluded and ended with Tessa and the community having to work through these feelings to re‐establish a sense of inclusivity.

Contrast the example above with Robert's feelings of exclusion at Hawthorne. Robert reported he was consistently excluded, despite the fact that he was quick to volunteer for community chores and usually adhered to community boundaries:

I've tried to join in more, I've tried to give more feedback if you like. But…I still don't feel a part of the group. I feel like there's everybody else and me. So…that gets me down, that I'm not a part of it.

Robert speculated during his interview that he was excluded because he was a male with a history of serial adultery: ‘They just see me as the bad guy and there's no answer for that’; and one client called Robert a ‘bastard’ out of earshot of staff. Rather than finding solidarity, like with Tessa above, the clients repeatedly resisted Robert's attempts to integrate.

At worst, marginalisation led to clients feeling isolated and distressed, with some willing to leave the TC. For Carl, the decision to leave Hawthorne came about after he felt isolated and chastised because of his verbal attack on a female client. Although the group discussed his conduct and voted to continue his membership during a community meeting ritual, he decided to voluntarily leave, saying he felt ‘emasculated’ and like a ‘predator’:

Then Carl's demeanour changes again. He sits back up, starts twitching and his voice raises. He starts to say again what he wants to work on but his voice really falters. Heather then asks how he feels about being voted in and he responds that he's really struggling. The group try to say they know he's anxious, but Carl says he feels like he has ‘cleavers’ hanging over him, like if he messes up again he would just be ‘beaten’. […] Carl then says, ‘sorry guys for wasting your time, I just can't do this’. (Powell, Day 12, 27/06/2013)

Carl had violated a community boundary and the client members felt a sense of indignation that they directed at him. Their response to him is in line with Collins's (2004: 49) assertion that ‘renegade insiders’ are treated more harshly than outsiders when ritual symbols, and social rules, are violated.

## Discussion

Although the boundaries between formal and informal therapeutic spaces within TCs are in some ways blurred, our study shows how clients with such programmes spend significant parts of their days outside of structured therapy and these spaces represent important sites for patterns of interaction. Drawing on Collins (2004), the patterns of interaction observed in these informal settings illustrate important IRCs, through which feelings of groups belonging and solidarity are developed and reinforced. Few studies specifically examine how these everyday interactions facilitate emotional and personal change. Our study shows how, within TCs, the experience of negative transient emotion can lead to high EE within the context of social interactions, and also lead to a shared sense of motivation and inclusion. Through our analysis we offer contributions to, first IRC theory, and also the social organisation of mental health practices.

Collins (2004) presents IRC theory as a model of social interaction that explains the mechanisms of successful, and repeated, rituals. Individuals repeat interactions that generate long‐lasting high EE, which is necessary for sustaining solidarity (Collins 2014). Long‐term negative emotions, including feelings of disunity (Freund 1990), are often seen as working against successful IRCs, as individuals avoid unpleasant or emotionally flat rituals. In our research, IRCs outside of structured therapy reveal the social mechanisms of solidarity and EE that sustain repeated interactions between clients. However, the Powell and Hawthorne case studies illustrate how these interactions often contained intense negative transient emotions, including anger and fear. Where these feelings might be expected to fracture unity, they were actually found to encourage and provide a foundation for a strong sense of solidarity, especially for individuals to actively support one another, and to enable newcomers to embody the community's emotional ideology and history (Summers‐Effler 2010). Whilst IRC theory acknowledges that negative emotional experiences can generate emotional energy and solidarity (Collins 2014), it does not fully explain how this is achieved. Our study suggests negative transient emotions contribute to sustained successful interaction rituals through the process of inclusivity. As interactions are connected through ‘chains’ (Collins 2004), negative feeling in one ritual continues to move between subsequent, integrated and complex interactions, which require members to transform these feelings into sources of solidarity. Within the case study communities, this complex process was more than sharing negative feelings; rather, because the emotional residue in one interaction would continue into other interactions, it involved continuously working together to challenge unhelpful narratives, such as Anna's feelings of isolation or Tessa's feelings of exclusion, in favour of a helpful group identity and sense of belonging.

**Figure 1:**
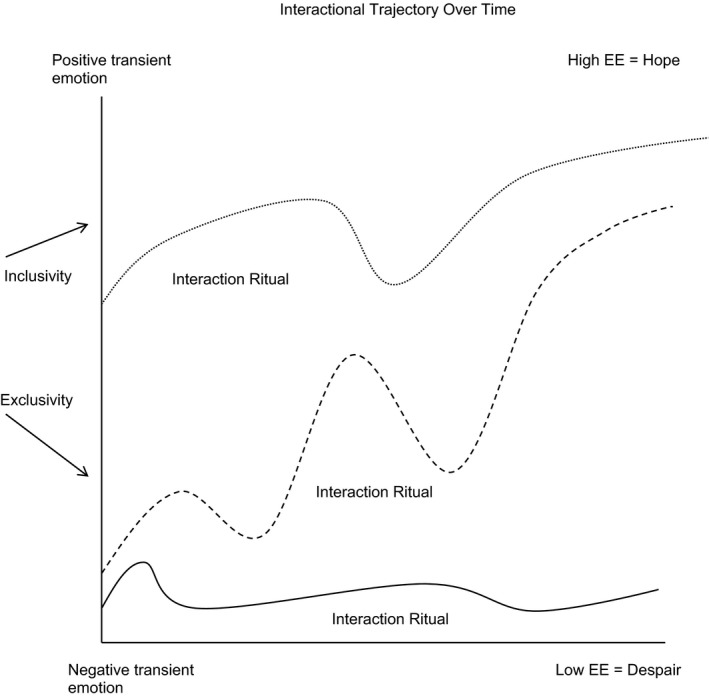
Interactional Trajectory Over Time

Establishing and maintaining solidarity in the TCs was not a straightforward process, particularly in the face of negative transient emotions. The key to understanding how negative emotions transform to EE may lie in Collins's (2014: 300) suggestion that failed rituals leave participants feeling ‘alienated’. As long as individuals continue to interact, like Christopher expressing his unhappiness, there is the opportunity to generate and maintain EE across the chain of rituals. Moreover, re‐establishing solidarity when interactions break down, including resolving tensions, appears to generate further feelings of security and trust (Helman 2007), resulting in increased confidence in the community, and in each person's role within it. When identity is shifted away from the individual, and one's negative history of interactions, to that of the group, then the individual is able to see oneself from the perspective of the collective (Summers‐Effler 2002). Identity comes not from the isolated individual, but from the collective formation of like‐minded others. Scott (2011: 242) makes a similar point by noting that when individuals ‘internalise’ the values of an institution, in this case a TC, the group's power is ‘benign’ and facilitates the ‘goal of self‐improvement’. The impact this shift has on negative emotions can be significant, as Summers‐Effler (2002: 50) explains, ‘the deviant emotions themselves have come to be associated with new sources for solidarity and emotional energy formed in collective identity’. EE can therefore be generated in the presence of highly intense, negative emotions. This strengthens IRC theory by showing how EE is not only dependent upon, or associated with, positive transient emotions. It suggests individuals will tolerate high amounts of negative feeling over long durations if they have a sense of belonging and hope in the community, as a form of emotional ‘payoff’ for feeling included. In other words, individuals can experience both transient negative feeling and high EE simultaneously. Thus EE is the affective expression of hope and confidence, based upon feelings of solidarity. These feelings can solidify in to feelings of hope and form an interactional trajectory; conversely, with low or no EE, feelings of despair and alienation can form with limited or no interaction. Figure 1 illustrates this process:

Collins (2014) argues that as an interaction unfolds, the emotional feeling may rise and fall, as represented by rituals’ patterns above. An interaction may begin with negative feeling, before providing the impetus and experience for solidarity. Each IRC has its own rhythm that moves towards an outcome of either high or low EE. Inclusivity and exclusivity exert a powerful push and pull momentum during a ritual that can generate high or low EE, respectively. This is shaped by the extent to which emotion, positive or negative, is shared as a basis for solidarity (Collins 2004). The character and context of the negative emotion must be mutual and inclusive in order to transform it into high EE. Conversely, as May's (2016) research highlights, being excluded from the group can have a significant detrimental impact upon one's sense of self, and one's sense of self in relation to others. Experiencing exclusivity, like Robert from Hawthorne, may generate feelings of alienation that could lead to negative feelings of depression and despair. Put differently, EE is not only a surge of energy that can spike feelings of confidence and hopefulness, it can be a steady source of energy consisting of underlying feelings of trust and belonging that motivates individuals to continuously engage in very difficult interactions over long periods of time. Interactions that build EE create a momentum of willingness to engage in future social interactions, whereas interactions that contain low EE generate avoidance of social encounters and may lead to social isolation.

In addition to expanding IRC theory, this paper extends discussions within mental health about the importance of ordinary moments in the therapeutic process. As suggested earlier, the application of IRC to healthcare has typically been concerned with understanding professional IRs, whereas our study focuses on the IRs of clients. This offers new insight into the ways everyday encounters outside of therapeutic or clinical episodes can contribute to the lived experiences of care and wellbeing. Though TCs are a distinctive form of therapy in valuing all forms of social encounters as potentially therapeutic, analysis of how times outside of therapy facilitate change is noticeably absent in the literature. These interactions are not neutral as they are important for building trust, resolving tensions, getting to know others, and contribute to the overall rhythm and life of an environment. Minimising the role of time outside of therapy risks undervaluing small but significant changes and may mask troubling encounters between clients, and between clients and staff.

Before we conclude, we reflect upon the limitations of our research. The focus of this study was on interactions outside of structured therapy. There are undoubtedly IRCs occurring within structured therapy that also contribute to the process of change. Indeed, this article does not suggest informal times could, or should, replace structured therapy or professional practice. It is unknown to what extent everyday social encounters facilitate personal change because of structured therapy and the expertise of staff members. The observations also focused on client member interactions and it unknown how staff members’ interactions may also facilitate inclusion and positive change for client members. Combining an analysis of times inside and outside of therapy, and staff interactions, may provide a deeper and more holistic analysis as to the role of all interaction rituals within TCs and other psychiatric units. Lastly, TCs are specialist units, and whilst the everyday interactions described occur in other settings (Quirk *et al*. 2006), exploring IRCs within other mental health environments would enable a richer application of IRCs within mental health.
